# Histopathology of Psoriatic Arthritis Synovium—A Narrative Review

**DOI:** 10.3389/fmed.2022.860813

**Published:** 2022-07-01

**Authors:** Catarina Tenazinha, Rita Barros, João Eurico Fonseca, Elsa Vieira-Sousa

**Affiliations:** ^1^Department of Rheumatology and Metabolic Bone Diseases, Hospital de Santa Maria, Centro Hospitalar Universitàrio de Lisboa-Norte, Lisbon, Portugal; ^2^Rheumatology Research Unit, Instituto de Medicina Molecular João Lobo Antunes, Faculdade de Medicina, Lisbon Academic Medical Center, Universidade de Lisboa, Lisbon, Portugal

**Keywords:** psoriatic arthritis (PsA), synovial membrane (SM), synovium, synovitis, spondyloarthropathy, inflammatory arthritis, histopathology

## Abstract

Psoriatic arthritis (PsA) is a phenotypically heterogeneous chronic inflammatory disease associated to type I major histocompatibility complex alleles whose complex pathogenesis is still not completely understood. The psoriatic synovium shares general features of chronic inflammation with rheumatoid arthritis (RA) and other arthritis, such as hyperplasia of the intimal lining layer, sublining influx of inflammatory cells and neoangiogenesis, but recognizing disease-specific histopathologic findings may help in diagnosis and definition of therapeutic targets. Available literature reports conflicting data regarding the extension of lining hyperplasia, that does not allow depiction from RA. Sublining inflammatory cells consist of T and B cells and macrophages, plasma cells, mast cells and follicular dendritic cells, with a higher amount of overall T, mast cell and IL-17 producing CD8+ T lymphocytes and lower proportion of plasma cells when compared to the rheumatoid synovium. The amount of synovium IL17+ CD8+ T cells correlates positively to measures of disease activity. Lymphoid follicles with characteristics of germinal centers have been identified, similar to the ones described in RA. Neoangiogenesis is more prominent in PsA but can also be an outstanding feature in some RA samples, and different molecules involved in the process appear to have different influence in each disease. IL-17 and IL-22 expression in the synovium does not allow depiction between diseases. Among other cytokines and molecules likely implicated in disease physiopathology, only IL-35 is demonstrated to be reduced in PsA when compared to RA.

## Introduction

Psoriatic arthritis (PsA) is a chronic inflammatory disease with high clinical heterogeneity that occurs the most commonly in patients with previously diagnosed psoriasis (Pso). It can involve joints, with heterogeneous patterns, and periarticular structures, such as the enthesis, tendon sheets, paratenons, but also the bone and the subcutaneous fat (1). Inflammation is considered to develop adjacent to bone insertions, affecting the enthesis and the synovium, and being associated with both processes of bone destruction and new bone formation. Clinical heterogeneity results from a wide range of severity and clinical features, that vary according to the tissues and body sites involved (2, 3).

Psoriatic arthritis pathogenesis is complex and detailed disease mechanisms are still unclear, with underlying polygenic and environmental predisposing factors. It is associated to type I major histocompatibility complex (MHC) alleles, with risk polymorphisms in genes related to interleukins (IL) 23 and 12, nuclear factor κB and tumor necrosis factor (TNF) that, when dysregulated, can promote the activation of inflammatory pathways (1). The study of psoriatic joint disease at the histopathology level has helped to understand disease mechanisms and may unravel new therapeutic targets and biomarkers, improving early diagnosis and treatment.

Available literature consists of either study reports or reviews of specific aspects, that often lack comparison to the most important synovitis counterpart of PsA, which is rheumatoid arthritis (RA). Herein we provide a descriptive and detailed description of the histopathologic findings concerning all levels of the synovial layer, aiming to identify what is known and what is yet to be understood, supporting physicians in biopsy interpretation and investigators in establishing questions.

## Overarching Findings in the Inflamed Synovium

Regardless of the underlying disease, chronically inflamed synovium displays three major histopathologic features:

1. Hyperplasia of the intimal lining layer. This occurs due to both accumulation of macrophages and proliferation of fibroblast-like synoviocytes (FLS).
2. Sublining influx of inflammatory cells. Infiltrating leukocytes, such as macrophages, dendritic cells, lymphocytes and mast cells, are activated and produce a vast amount of pro-inflammatory and damage mediators that contribute to synovitis as well as to cartilage and bone destruction. Lymphocyte infiltration can be described in three different patterns: diffuse infiltration, B and T cell aggregates, and ectopic lymphoid aggregates with prototypical features of germinal centers, such as the presence of follicular dendritic cells and high endothelial venules.
3. Sublining neoangiogenesis. Vascularization is increased due to endothelial activation in the synovial sublining layer (4).

Inflammatory mediators, the complement cascade and other proteins are involved in the genesis and perpetuation of the inflammatory process, with different roles and importance according to the underlying disease.

### Hyperplasia of the Intimal Lining Layer

Lining hyperplasia has been regarded as the best evidence of FLS expansion. The first important study attempting to characterize the psoriatic synovium evaluated samples obtained from biopsy of large inflamed joints in PsA patients, by both light and electron microscopy. The synovial lining layer was described as composed by synoviocytes type A (phagocytic) and type B (synthetic), with minimal to non-existent synoviocyte hyperplasia and hypertrophy in most patients, including those with longer disease duration. No abnormal ultrastructural abnormalities were identified by electron microscopy (5, 6). Following studies described indeed mild hyperplasia of the psoriatic synovium, as opposed to the significant hyperplasia found in the rheumatoid synovium (6–8). Interestingly, higher numbers of lining fibroblasts have been found in the synovium of patients with undifferentiated arthritis who progressed to PsA, compared to that of patients who ended up diagnosed with RA (9). Synovial lining layer thickness varies in psoriatic synovium, ranging from 2 to 5 cells, with an average thickness of 3 cells (7, 8, 10). Ceponis et al. hypothesized the existence of differences in apoptotic patterns of inflammatory cells in the synovium stroma as one of the underlying causes of lining thickness disparity between diseases (10). Lining layer cell apoptosis is indeed increased in psoriatic synovium, but its extent and pattern do not differ significantly from those observed in rheumatoid synovium (10). The presence of cellular debris and fibrin has also been described, as have the presence of hypertrophied synoviocytes with long cytoplasmatic processes that extend into the joint space, whose significance remains unknown but are probably related to the inflammatory process, regardless of its underlying mechanism (6, 11).

Infiltration by cells of the monocyte/macrophage linage, as given by the pan-macrophage marker CD68+, contributes to the lining thickness, and was suggested to be present in a lesser extent in PsA than in rheumatoid synovium, which would also be reflected in a lower expression of the cytokines TNF, IL-1beta and IL-15 (8, 10). Yet, more recent evidence failed to demonstrate this difference (7, 12, 13). In fact, also van Kuijk did not find differences not only in the macrophage/monocyte linages, but also in the lining thickness, after matching patients according to disease duration and drug exposure (14). Regardless of its extension, a reduction in macrophage counts in both lining and sublining layers is observed in response to anti-TNF therapy in PsA synovium (15).

Moreover, neither differences in lining layer infiltrates nor thickness between short and long disease duration, nor between polyarticular and oligoarticular psoriatic disease, have been found (6, 13).

### Sublining Influx of Inflammatory Cells

**FIGURE 1:**
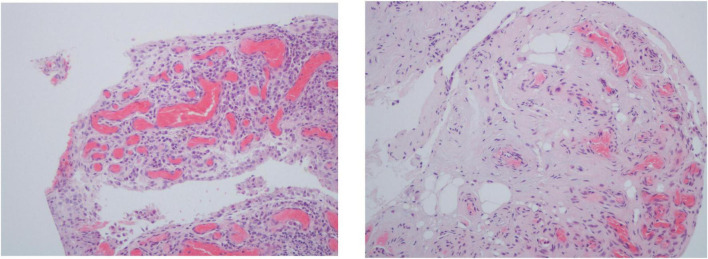
Histologic features of psoriatic arthritis synovium including 2–3 thickness cell lining layer hyperplasia, high vascular density and perivascular infiltration (hematoxylin-eosin, 200×).

Inflammatory cells infiltrate in PsA synovium are mostly perivascular but focal aggregates and diffuse infiltrates can also be seen. The amount and extent of the infiltrates vary from mild to marked (Figure 1) (5, 6).

#### Inflammatory Cells

The analysis performed by immunohistochemistry demonstrates the presence of T (CD3+), B (CD20+), macrophage (CD68+), plasma (CD138+), mast (CD117+), and follicular dendritic (CD21+) cells in the inflammatory infiltrates. Overall, T lymphocyte and plasma cell amounts are lower than those observed in rheumatoid synovium (9, 10, 12–14, 16). The proportion of CD4+/CD8+ T lymphocytes, and CD4+ and mast cell infiltration, however, appears to be higher than in RA (12, 17). Caution in interpreting these findings is needed as CD4 is also expressed by macrophages (14). Mast cells are increased in spondyloarthritis (SpA) as a whole and account for the majority of the IL-17 expression in the synovium, in contrast to RA. This has been demonstrated for PsA and other SpA by comparison to RA, but no comparative studies have been made among SpA subtypes (18). Because plasma cells are found in higher amounts in seronegative RA and not in PsA, and mast cells are increased in PsA to a larger extent in comparison to RA, the ratio of both cell scores (mast and plasma cells) has been proposed as a possible differentiator between both diseases (12, 19). Along with lining fibroblasts, infiltrates richer in mast cells in undifferentiated arthritis have recently been suggested as predictors of progression to PsA (9). Also, B cells express surface type II MHC and co-stimulatory molecules that provide them antigen presenting-cell features, and these phenotypic changes, although also demonstrated in psoriatic synovium, involve a larger molecule repertoire in rheumatoid synovium (20). Nevertheless, no differences have been identified regarding the number of CD20+ B cells, follicular dendritic cells, and macrophages between RA and PsA synovium (8, 12, 13, 17).

T lymphocyte and macrophage infiltrates also respond to anti-TNF therapy in both diseases, but a greater cell depletion is observed in RA patients in remission compared to PsA counterparts, suggesting higher residual inflammatory activity in the later.

Similarly to the synovial lining layer, Kruithof et al. could not find differences in sublining inflammatory infiltrates between poly and oligoarticular disease phenotypes (13).

#### Lymphoid Follicles

Lymphoid follicles have primarily been studied in RA (21), and Canete et al. were the first to study lymphoid neogenesis in PsA. Their work described a prevalence of around 50% of ectopic lymphoid neogenesis in the synovium of PsA patients, with cell organization and molecular features similar to those of germinal centers, consisting of T/B cell segregation, presence of addressin-positive high endothelial venules (HEV-PNAd+) and expression C-X-C Motif Chemokine Ligand 13 (CXCL13), C-C Motif Chemokine Ligand 21 (CCL21) and C-X-C Motif Chemokine Ligan 12 (CXCL12) chemokines. Both the number of T/B cell segregated lymphoid infiltrates and overall germinal center molecular expression are increased in accordance to the lymphoid follicle size (radial cell count of lymphoid aggregation) (16). In RA, lymphoid aggregation is related to serum rheumatoid factor positivity. Rheumatoid synovium does present features of antigen-specific lymphocyte activation, and at least one candidate T cell auto-antigen has been identified in a large cohort of patients, with high disease specificity (22, 23). Yet lymphoid aggregates are found to be non-specific and reflect the degree of local inflammation rather than the magnitude of local antibody production (21).

Expression patterns of peripheral lymph-node HEV-PNAd+, CXCL13, C-CCL21, and CXCL12 in PsA match the ones that have previously been described in RA, and do not correlate to any clinical features. It has been demonstrated, however, that lymphoid follicular depletion after anti-TNF treatment is associated to sustained clinical remission in PsA (16).

#### Fibrosis

The PsA synovial sublining is rich in collagen fibers. Fibrosis is mainly perivascular, its degree is highly variable and, even though it tends to be a prominent feature in advanced disease, increased areas of fibrosis in the sublining layer adipose tissue and joint recess are unexpectedly great in some patients with short disease duration, and surprisingly scarce in long-lasting synovitis (5, 6). The presence of fibrosis is also a well-established feature of the synovial tissue of some RA patients (24).

### Synovial Sublining Vascularization

Of the three major histological hallmarks of synovial inflammation, only vascularization appears to be, overall, different between RA and SpA (4). Although neovascularization occurs in both diseases, in early and established stages, vascularization is increased in both SpA as a group, and PsA in particular, compared with RA. Marked synovial vascularity is a relevant finding in the synovial tissue of most PsA patients.

**FIGURE 2:**
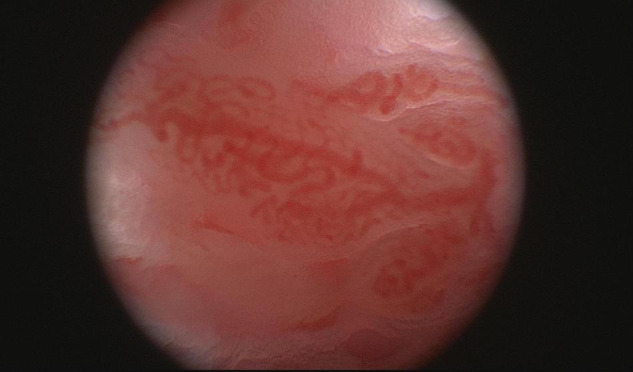
Macroscopic aspects of the synovium of a psoriatic arthritis patient, showing a tortuous and bushy vascularization.

Macroscopic studies by arthroscopy in patients with PsA and RA demonstrated different vascular patterns, describing tortuous, bushy vessels in the former and predominantly straight, branching vessels in the latter (Figure 2). Because some RA patients do display a mixed vascular pattern, the vascular findings described in psoriatic synovium cannot be considered specific of this entity but they are remarkably more prominent in PsA than in RA (14, 25, 26). Straight vessels are also found in other subsets of SpA (25). Psoriatic skin lesions present changes in vascular morphology that resemble the ones present in PsA joints and on nailfold videocapilaroscopy (27), highlighting angiogenesis as a hallmark event in PsA. Although macroscopic evaluation by arthroscopy suggests different vascular patterns, in agreement with microscopic vascular findings, the correlation between both is weak (25).

Histologic synovial vascularity in PsA is characterized by increased thickening of the vascular wall and prominent endothelial lining layers of capillaries and small arteries, due to endothelial cell swelling – dilated rough endoplasmic reticulum and intracellular oedema were observed on electron microscopy—with polymorphonuclear, lymphocyte, monocyte, and plasma cell inflammatory infiltration in the media and adventitia (5, 13, 25, 28). Even though Zhang et al. corroborated the presence of the marked vascular changes previously described in psoriatic synovium, they failed to confirm the presence of endothelial intracellular oedema, therefore suggesting that this feature might not be characteristic of all PsA patients. In this study, two findings were reported: very small dense deposits on the vessel walls, resembling immune complexes, and microparticles, membrane-bound vesicles on endothelial cells, known to regulate thrombosis, angiogenesis, vascular reactivity, and inflammation (6).

Overexpression of vascular markers has been shown to occur in psoriatic synovium, namely vascular endothelium growth factor (VEGF), von Willebrand’s factor (vWF), integrin αVβ3, and basic fibroblast growth factor (bFGF), along with upregulation of vascular cell adhesion molecule 1 (VCAM-1), intercellular adhesion molecule 1 (ICAM-1) and E-selectin (29). Tie-2 is involved in angiogenesis upregulation through the interaction of its activated form with its ligands angiopoietin-1 (Ang1) and angiopoietin-2 (Ang2), the three of which are overexpressed in psoriatic and rheumatoid synovia, where they contribute to inflammation by inducing inflammatory gene expression in endothelial cells, FLS and macrophages (30, 31). VEGF and Ang2 are overexpressed in early disease stages in both PsA and RA but their expression is higher in the perivascular and lining layer regions of the psoriatic synovial membrane, with low levels of Ang1. On the other hand, in the synovial tissue of early RA, there is upregulation of the Ang1/Tie-2 axis to a higher degree than what is observed in psoriatic synovium (32). Furthermore, little to no endothelial leukocyte adhesion molecule 1 (ELAM-1) expression has been found on endothelial cells of psoriatic synovium, in contrast to RA, where its expression is intense and widespread. As for the expression patterns of ICAM-1, VCAM-1 and E-selectin, as well as metalloproteinases (MMP) 1, 3, 13 and anti-tissue inhibitor of MMP1, no differences have been described between PsA and RA (8, 13, 14). ICAM-1 was identified in endothelial, lining layer and mononuclear cells within the stroma in both diseases. Neither disease expressed VCAM-1 on its vascular endothelium, with VCAM-1 being expressed only in lining layer cells and on a few cells in the inflammatory infiltrates resembling dendritic cells (8). Surprisingly, differences in vascular markers VEGF, vWF, alfavbeta3, and bFGF were also not found (14).

MicroRNA-125a (miRNA-125a) is an angiogenic down regulator whose expression appears to be reduced in psoriatic synovium. The levels of miRNA-125a were demonstrated to be inversely correlated to the degree of endothelial activation (33).

No differences regarding vascularization in the synovial membrane have been demonstrated between oligo and polyarticular disease phenotypes of PsA (14).

### Inflammatory Mediators

Th17-related genes are upregulated and the expression of IL-17 increased in psoriatic synovium, but Van Baarsen et al. failed to find differences in the levels of IL-17A, IL-17F and their receptors among RA, PsA and osteoarthritis (OA) patients. Interestingly, they reported interindividual variety of degree of expression inside each of these three specific diseases (34, 35). The overexpression of IL-17 is higher in psoriatic skin lesions than in synovial tissue, potentially explaining the better response of skin lesions to anti-IL17 therapy (36). Menon et al. also demonstrated that IL-17+ CD8+ T cells are present at increased levels in psoriatic synovium when compared to paired samples of PsA peripheral blood and synovial samples from patients with RA, and that they are correlated to serologic (C-Reactive Protein and Erythrocyte Sedimentation Rate), clinical (Disease Activity Score 28) and imaging (power Doppler ultrasound score) features of PsA activity and erosive disease status (37).

IL-22 belongs to the Th17 pathway and its receptor (IL-22R) is expressed in FLS in psoriatic synovium, but also in rheumatoid and osteoarthritic synovial tissue. Activation of IL-22R in FLS by its ligand, produced by activated T-cells, leads to FLS proliferation (38). In psoriatic skin plaques, IL-22 expression is high when compared to healthy skin and appears to have a role in the pathogenesis of the disease as it is associated to higher expression of S100A7, S100A8, S100A9, and MMP1 by keratinocytes (39).

Psoriatic synovium expresses elevated levels of Th1 cytokines, that include IL-2, TNF, IFN-gamma, IL-1beta, IL-6, and IL-18. However these observations are not significantly different from what is reported in rheumatoid synovium (14, 40).

IL-36 is a pro-inflammatory cytokine family, belonging to the larger IL-1 family, whose role in psoriatic skin disease is well established (41–43). Its function in joint disease, however, has just begun to be studied, and it has been shown to be expressed in large amounts in psoriatic synovium and to be related to poorer response to conventional synthetic disease modifying anti-rheumatic drug therapy (44).

IL-9 drives pannus formation by FLS activation and is therefore closely related to joint damage (45). Both IL-9 and its receptor (IL-9R) are strongly expressed in leukocytic infiltrates of the lining layer of psoriatic and rheumatoid synovia, in a higher amount than that observed in OA (45–47). IL-9 is produced by polarized Th9 cells and fibroblasts, present not only in the synovium, but also in the peripheral blood and sub-clinically inflamed gut. IL9 drives T-cell activation and IL-6, IL-8, and MMP3 production, as well as FLS survival and proliferation, by inducing phosphorylation of kinase signal transducer and activator of transcription (STAT) 3. Peripheral blood levels of Th9 cells correlate with disease activity and are reduced after treatment with anti-TNF or anti-IL17 drugs (45, 47, 48).

Several other cytokines have been shown to be highly expressed in psoriatic synovial tissue. IL-41 is markedly expressed in both psoriatic and rheumatoid synovia, and is induced by TNF and IL-17 (49). In addition, IL-15 is upregulated in synovial tissue in PsA patients. Its role in the arthritic process is related to natural killer cell activation (50). Finally, IL-20 is expressed in CD68+ macrophages and CD55+ synoviocyte-like fibroblasts both in psoriatic and rheumatoid synovium. In PsA it correlates to synoviocyte-like cell scores, but was not reduced by anti-IL20 therapy (51).

On the contrary, IL-35 and IL-8 are only moderately expressed in psoriatic synovium. Although IL-35 belongs to the IL-12 family, one of the well-established therapeutic targets in PsA and Pso, its expression in synovial tissue in PsA is significantly inferior to that observed in RA (52). IL-8 was more expressed in the synovial lining cells and in a lesser extent by the perivascular cells in psoriatic synovium (28).

### Complement and Other Proteins

C5a-receptor positive cells (mainly neutrophils and macrophages) are present in the synovium of PsA, RA and OA patients, but not in that of non-inflammatory controls (53).

Pro-C2 and C-Col10 are molecular markers for the synthesis and turnover of collagen type IIB and X that were found to be increased in psoriatic synovium when compared to healthy controls (54). Osteoactivin, an anabolic factor involved in osteoblast differentiation and function, is also overexpressed (35, 55).

CD248 is a transmembrane glycoprotein demonstrated to be expressed in stromal fibroblast-like and perivascular activated cells in synovial tissue of PsA and RA patients, but not in that of healthy subjects. In animal models, it correlates with arthritis severity (56).

The granulocyte calcium-binding protein S100A12, previously suggested as a potential disease marker, is not useful in distinguishing PsA from RA and other SpA (8, 13, 57).

Phospho-signal transducer and activator of transcription (pSTAT) 1 and 3 of the Janus Kinase signaling pathways are overexpressed in synovicyte-like fibroblasts from synovial tissue of PsA and RA patients, and also in keratinocytes from psoriasis patients (58).

No intracellular citrullinated proteins are observed in PsA. 527 MHC-HC gp39 peptide complexes are also not found (13). 528 Tumor suppression p53 protein is poorly expressed in the 529 psoriatic synovium, as opposite to its high expression in both 530 lining and sublining layers of rheumatoid synovium (17).

## Enthesitis Histology

Biopathology at the synovio-entheseal level is complex. The interdependence between synovial and entheseal inflammatory processes is not fully understood and the consistency of their co-existence, although well demonstrated in the knees and distal interphalangeal joints of SpA patients, is less clear in other joints (3, 59, 60). Data on entheseal inflammation in humans is limited given the difficulty in obtaining tissue from patients with enthesitis, and therefore most of the current knowledge regarding enthesitis histopathology derives from animal models (61). Laloux and colleagues identified oedema and inflammatory infiltrates in the bone marrow part of enthesis of SpA patients (only one with PsA), that were present more often and in a greater extent compared to those observed in RA, whose inflammation was predominantly located in the soft tissue rather than in the bone marrow component. The infiltrates in SpA enthesis presented increased numbers of all cell types but the difference was more marked in respect to T lymphocytes, particularly of the CD8+ type (62). A study by McGonnagle et al. of five SpA patients, that did not include any patients with PsA, described an inflammatory infiltrate high in macrophage counts, but lymphocytes were not increased, and vascularity was altered, demonstrated by increased CD34+ staining (63).

## Contributions to Precision Medicine

Treatment decision in PsA is currently, and similarly to other rheumatic diseases, grounded in features of a cluster of clinical domains and on pharmacoeconomic analysis. Studies in RA are paving the way for treatment choice strategies based in synovial tissue biomarkers (64, 65). In PsA, Miyagawa and colleagues were pioneers, demonstrating a higher efficacy of tailored biologic disease modifying anti-rheumatic drug (bDMARD) treatment to helper T cell phenotypic differences in peripheral blood, when compared to standard bDMARD (66). A panel of proteins that include S100A8, S100-A10, Ig kappa chain C, fibrinogen-α and γ, haptoglobin, annexin A1 and A2, collagen alpha-2, vitronectin, alpha-1 acid glycoprotein, cofilin, prolargin, 14-3-3 protein epsilon and clusterin isoform 1, identified in the synovial tissue samples of PsA patients, can predict response to anti-TNF therapy in PsA (67). Among the panel, S100A8, which has a proven role in inflammation regulation and immune response, and is highly expressed both in PsA and RA synovial tissue, is, alone, the best predictor of response to anti-TNF therapy (67, 68). Identification of germinal centre-like structures admits a theoretical role for B cell-depleting therapies in PsA, but studies in SpA have been disappointing (69, 70). Having been reported a lack of difference on IL-6 levels between PsA and RA, a response to anti-IL-6 receptor inhibitor could also be expected, but failed to excel in the clinical set (71).

Even though therapies directed to molecules found to be overexpressed turned out to be successful, such as anti-TNF and Janus kinase inhibitors, both in RA and PsA, data from failed clinical trials have taught us that targeting (apparent) key pathways may not always result in obvious clinical benefit (72, 73). Also, these pathogenic mechanisms are complex and not completely understood, as previously stated, and crosstalks among signalling pathways may result in a given molecule weighing differently for a common histologic outcome in independent diseases.

## Discussion

In this review we describe histologic features of psoriatic synovium, underlying the most consistent findings from the literature in what concerns cellular and molecular contributors, focusing on the differential diagnosis with RA. A moderate to marked lining layer hypertrophy dependent on FLS proliferation and macrophages infiltration in psoriatic synovium has been described, but this feature is far from allowing distinction between synovial tissue from RA and PsA patients. Both in PsA in RA the inflammatory infiltrates in the sublining layer are composed of B cells, T cells, plasma cells, macrophages, mast cells and follicular dendritic cells, as well as ectopic lymphoid follicles with the only consistent difference established to date between both diseases in this regard appearing to be higher numbers of mast cell counts in PsA, and of plasma cells in RA. Also, a more marked histopathologic response to anti-TNF therapy is observed in RA than in PsA, with higher depletion of T cell and macrophage infiltrates observed in RA during remission on treatment. Overall, IL-17 expression is similar between diseases but a subset of CD8+ IL-17 producing cells is found more frequently in PsA and correlates to disease severity. IL-35 expression is significantly reduced in psoriatic synovium, with no other differences regarding cytokines being consistently identified. Altered vascularization is a prominent feature in PsA, but is not by itself sufficient to distinguish both diseases. Yet, vascular changes appear to have different underlying upregulated pathways, with predominant role of Ang2 in PsA and Ang1 in RA. Altogether, effective psoriatic synovium specific diagnostic biomarkers are still lacking, underlining a clear unmet need in this field.

## Author Contributions

All authors listed have made a substantial, direct, and intellectual contribution to the work, and approved it for publication.

## Conflict of Interest

The authors declare that the research was conducted in the absence of any commercial or financial relationships that could be construed as a potential conflict of interest.

## Publisher’s Note

All claims expressed in this article are solely those of the authors and do not necessarily represent those of their affiliated organizations, or those of the publisher, the editors and the reviewers. Any product that may be evaluated in this article, or claim that may be made by its manufacturer, is not guaranteed or endorsed by the publisher.
